# Arabidopsis plants deficient in constitutive class profilins reveal independent and quantitative genetic effects

**DOI:** 10.1186/s12870-015-0551-0

**Published:** 2015-07-11

**Authors:** Kristofer J. Müssar, Muthugapatti K. Kandasamy, Elizabeth C. McKinney, Richard B. Meagher

**Affiliations:** Genetics Department, Davison Life Sciences Building, University of Georgia, Athens, GA 30602 USA

**Keywords:** Actin, Profilin, *Arabidopsis thaliana*, Constitutive, Functional redundancy

## Abstract

**Background:**

The actin cytoskeleton is involved in an array of integral structural and developmental processes throughout the cell. One of actin’s best-studied binding partners is the small ubiquitously expressed protein, profilin. *Arabidopsis thaliana* is known to encode a family of five profilin sequence variants: three vegetative (also constitutive) profilins that are predominantly expressed in all vegetative tissues and ovules, and two reproductive profilins that are specifically expressed in pollen. This paper analyzes the roles of the three vegetative profilin members, PRF1, PRF2, and PRF3, in plant cell and organ development.

**Results:**

Using a collection of knockout or severe knockdown T-DNA single mutants, we found that defects in each of the three variants gave rise to specific developmental deficiencies. Plants lacking PRF1 or PRF2 had defects in rosette leaf morphology and inflorescence stature, while those lacking PRF3 led to plants with slightly elongated petioles. To further examine these effects, double mutants and double and triple gene-silenced RNAi epialleles were created. These plants displayed significantly compounded developmental defects, as well as distinct lateral root growth morphological phenotypes.

**Conclusion:**

These results suggest that having at least one vegetative profilin gene is essential to viability. Evidence is presented that combinations of independent function, quantitative genetic effects, and functional redundancy have preserved the three vegetative profilin genes in the Arabidopsis lineage.

**Electronic supplementary material:**

The online version of this article (doi:10.1186/s12870-015-0551-0) contains supplementary material, which is available to authorized users.

## Background

Actin Binding Proteins (ABPs) facilitate rapid remodeling of the actin cytoskeleton by regulating the unpolymerized (G-actin monomers) and polymerized (F-actin filaments) actin (ACT) equilibrium [1]. Actin-ABP interactions regulate such processes as stress response, cell signaling, transcription, cytokineses, cell locomotion, organelle positioning and movement, nuclear transport, maintenance of cell size, shape, and polarity, and organ development [2–8].

Profilins (PRFs) are small (12–15 kDa), ubiquitously expressed, monomeric ABPs that have been identified in organisms ranging from most protists and all fungi to all higher plants and animals examined [9]. Originally, profilin was shown to specifically bind G-actin (globular actin) and was thought primarily responsible for G-actin sequestering in cells [10]. Recently, profilin has also been found to inhibit the spontaneous polymerization of actin filaments by forming a 1:1 complex with G-actin, thereby lowering ATP-G-actin steady-state concentrations. Once actin barbed ends (+ end of actin polarity) become blocked by capping proteins, profilin begins to sequester G-actin from pointed-end polymerization [11].

However, extensive research has shown that they also play a complex role in the formation of F-actin (filamentous actin) through the replenishment of the ATP-actin monomer pool via catalyzing the exchange of ADP for ATP on Actin [12]. While profilin does not bind F-actin directly, profilin-ATP-G-actin complexes are essential for rapid filament assembly [13]. Although profilin-bound actin monomers cannot add to pointed ends of actin filaments, they have been shown to elongate filament barbed ends at approximately the same rate as free actin monomers [14, 15]. This would lead us to believe that profilin might be facilitating rather than inhibiting polymerization [16]. This idea is further strengthened by results indicating that profilin could lower the critical concentration of actin needed to drive polymerization [17]. While the role of profilin in actin mechanics and signaling has been explored in detail, its role in development is tissue and development is poorly understood.

Higher plant and animal profilins are encoded by small gene families, which are independently evolved from ancestral profilins and exhibit distinct tissue and organ-specific expression patterns throughout development [18, 19]. The vegetative and pollen-specific classes of plant profilins show significant amounts (~25 %) of amino acid sequence divergence. These profilin classes are functionally distinct in their interaction with vegetative and reproductive class actins [20, 21]. Sequence conservation among all profilins among monocots and dicots reveals that vegetative profilins in monocots and dicots are more similar to each other than they are to their own reproductive profilin counterparts. It has been suggested that reproductive and vegetative class profilins coevolved with the vegetative and reproductive actins early in land plant evolution, well before the split between monocot and dicot angiosperms [22, 23].

In *Arabidopsis thaliana*, there are five profilin genes (PRF1-PRF5). PRF1, PRF2, and PRF3 are vegetative, being constitutively expressed throughout all vegetative tissues and in ovules, but not in pollen, and were originally classified as “vegetative” or “constitutive profilins”. PRF4 and PRF5 are classified as reproductive profilins and are predominately expressed in mature pollen [24]. The three vegetative proteins share 90 % sequence identity, whereas the vegetative and reproductive classes share 70-75 % sequence homology [25]. While the expression levels of PRF4 and PRF5 are essentially indistinguishable, the vegetative profilins exhibit a widely varying range of expression. In young leaf tissue, PRF2 is the most highly expressed, PRF1 is only expressed at moderate levels (~40 % of PRF2 levels), and PRF3 is weakly expressed (~12 % of PRF2 levels). Despite varying in their amounts of expression, initial evidence suggests that PRF1, PRF2, and PRF3 are expressed in a similar spatial pattern [26].

While there has already been some research depicting the function of these *Arabidopsis* profilins, their effects on overall plant development still remain a mystery. Previous analysis has shown that a partial knockdown (RNA and protein levels 50 % of WT) of the vegetative profilin, PRF1, results in altered seedling development, elongated hypocotyls, loss of light regulation, as well as defects in root hair development, flowering time, cell elongation, and overall cell shape maintenance [27, 9]. However, due to the leaky nature of the mutants being examined, these phenotypes were not overwhelming, suggesting that complete knockouts as well as double and triple knockouts will need to be established and dissected in detail. Biochemical analysis and localization observations have shown that PRF1 has a higher affinity for binding poly-L-proline and G-actin than PRF2, and that while PRF1 is more likely associated with filamentous actin, PRF2 localizes to polygonal meshes resembling the endoplasmic reticulum [28]. A detailed functional analysis of PRF3 has not been previously reported.

We describe here, using various knockout transfer DNA (T-DNA) insertion mutants and RNA interference (RNAi) knockdown plants in multiple combinations, the roles of the three *Arabidopsis* vegetative protein variants in cell, tissue, and organ development. The creation of double mutants showed more extreme combinations of the single mutant phenotypes, while knocking down all three profilins showed the most drastic dwarfed phenotypes as well as problems with lateral root initiation and growth. These data indicate the quantitative genetic effects and independent roles for the three vegetative profilins.

## Results

### Vegetative profilin single mutants show defects in leaf and inflorescence development

**Fig. 1:**
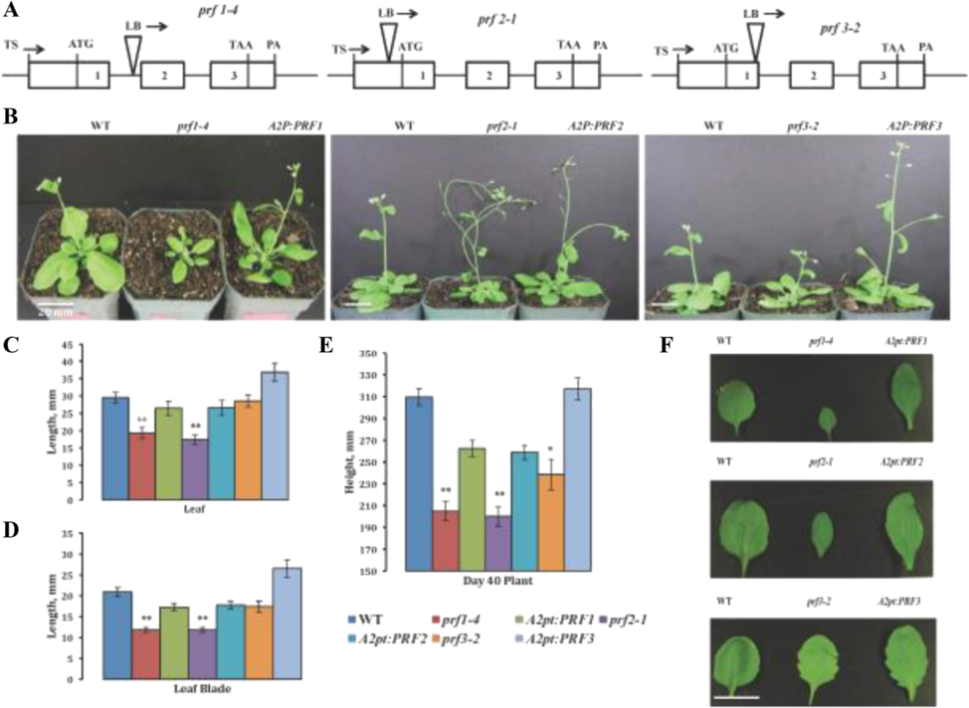
Analysis of mutants defective in individual vegetative profilins. **a** Schematic drawings indicating the location of each T-DNA insertion in mutant plants *prf1-4*, *prf2-1*, and *prf3-2*. **b** Visualization of adult plant morphological phenotypes of profilin T-DNA mutants, wild type (WT), and each mutant (*prf1-4*, *prf2-1*, *prf3-2)* complemented with the appropriate transgene (*A2P:PRF1, A2:PRF2,* and *A2:PRF3*, respectively). Pictures were taken 4 weeks (4w) after seed germination. **c** Leaf length for single vegetative PRF T-DNA mutants and their complemented lines. **d** Leaf blade length for single vegetative PRF T-DNA mutants and their complemented lines. **e** Mature plant height for single vegetative PRF T-DNA mutants and their complemented lines. **f** 4 week (4w) leaf morphology pictures of individual leaves from single PRF T-DNA mutants, WT, and complement lines. Leaf measurements (C and D) were taken on day 28 (4w) (*n* = 52) following seed germination, while plant height measurements (E) were taken on day 40 (~5 ½ w) (*n* = 30). All measurements are in mm. Error bars represent +/- 1 SD. **p-value < 0.001. *p-value < 0.05

Initially, we characterized single T-DNA insertion mutants for PRF1, PRF2, and PRF3. The *prf1-4* allele has an insertion in the first intron 74 bp upstream of the second exon, *prf2-1* has an insertion 113 bp upstream of the translational start site in the promoter, and *prf3-2* has an insertion at the end of the first exon (Fig. 1a). To ensure that the resulting mutant phenotypes were indeed caused by these specific insertions, we constructed lines that were complemented by overexpressing endogenous PRF1, PRF2 or PRF3 cDNAs, respectively, under the control of the constitutive Actin2 promoter and terminator (A2pt). Two or more independent transgenic complementation lines were analyzed.

The mutant allele’s *prf1-4* and *prf2-1* displayed significant visible defects in rosette leaf and inflorescence development at day 28 after germination as shown in Fig. 1b. At this stage *prf3-2* plants appeared to have leaves relatively normal except for slightly elongated petioles. The *prf1-4* and *prf2-1* plants developed leaves that are significantly shorter in total length, width, and blade length (Fig. 1c, d and f). All three mutant alleles produced plants that were shorter in overall plant height (Fig. 1e), with inflorescences appearing obviously less physically stable in *prf2-1* than that of WT (Fig. 1b). Pictures of these mutant plants at other stages of development may be seen in the (Additional file 1: Figure S1).

**Fig. 2:**
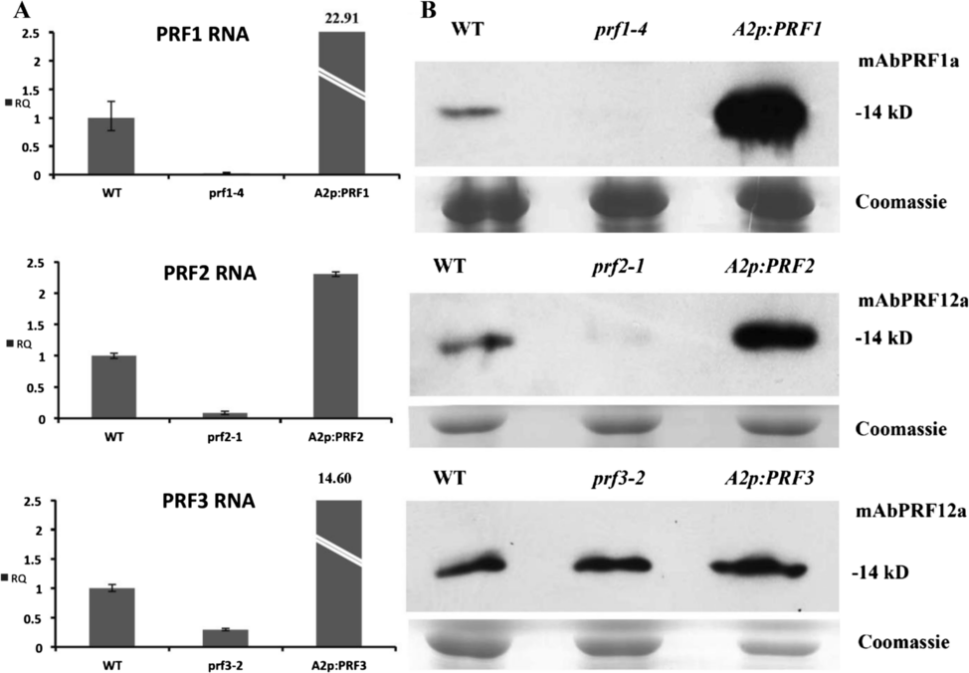
Analysis of profilin RNA and protein expression for vegetative PRF single T-DNA mutants and complement lines. **a** The Relative Quantities (RQ) of *PRF1* RNA for WT, *prf1-*4, and *A2p:PRF1* plants were determined by quantitative Real Time PCR (qRT-PCR). The RQ of *PRF2* RNA for WT, *prf2-1*, and *A2p:PRF2* plants and the RQ of *PRF3* RNA for WT, *prf3-2*, and *A2p:PRF3* plants are also shown. Error bars represent +/- 1 SD. **b** Profilin protein expression was examined by Western blot analysis: WT, *prf1-4*, and *A2p:PRF1* plants using the PRF1 specific monoclonal antibody mAbPRF1a; WT, *prf2-1*, and *A2p:PRF2* plants using the PRF1 and PRF2 specific monoclonal antibody mAbPRF12a; WT, *prf3-2*, and *A2p:PRF3* plants using the PRF1 and PRF2 specific monoclonal antibody mAbPRF12a, which also has weak affinity for PRF3. Coomassie stained gels showing rubisco protein expression are located beneath each blot to show equal loading across lanes. All samples were taken from 4w old leaf tissue

The levels of profilin RNA and protein in these lines were determined using qRT-PCR and western blot analysis, respectively. The monoclonal antibody mAbPRF1a reacts strongly and specifically with PRF1, while mAbPRF12a reacts strongly with PRF1 and PRF2 and only modestly with PRF3 [19]. qRT-PCR and western blot analysis revealed that these mutants had very little or no detectable RNA or protein expression (Fig. 2a-b). Although, based on the location of the insertion, *prf1-4* is probably not a null allele and may produce some level of RNA, the PRF1 protein expression was below our detection limit. The *prf2-1* line has very little vegetative profilin protein and similarly low *PRF2* RNA. The *prf3-2* line does not show a reduction in protein but a substantial reduction in RNA. Based on the site of insertion prf2-1 and prf3-2 are most likely null for functional profilin protein expression. We also demonstrated that the complement lines contained much higher levels of RNA and protein than WT (Fig. 2a-b). While these complemented lines appear to bolt slightly earlier than WT (Fig. 1b), no statistically significant phenotypes were observed in these lines overexpressing any of the three vegetative profilins (Fig. 1c-e).

To independently confirm the major phenotypes produced by deficiencies in PRF1 and PRF2, we created single RNAi silencing epialleles (*PRF1*-RNAi and *PRF2*-RNAi). We developed a new efficient method for constructing RNAi genes that expressed simple stem-loop structures with tetra-adenosine in the loop, to silence RNA expression (see Methods). These stem-loop structures were designed to target and silence the 3′-UTR of each gene. A series of epiallelic lines expressing these constructs were isolated. Most of the lines produced morphological phenotypes indistinguishable from the T-DNA insertion mutants. The resulting measurements of leaf length, width, and plant height for selected lines are presented in Additional file 2: Figure S2. qRT-PCR of these epiallelic lines revealed that PRF1 and PRF2 transcript levels were less than 10 % of WT (Additional file 2: Figure S2). Unfortunately, we were unable to create a successful, clean RNAi line for PRF3 using various methods.

### Vegetative profilin double mutants and double/ triple RNAi lines show more severe

#### Effects on development

**Fig. 3:**
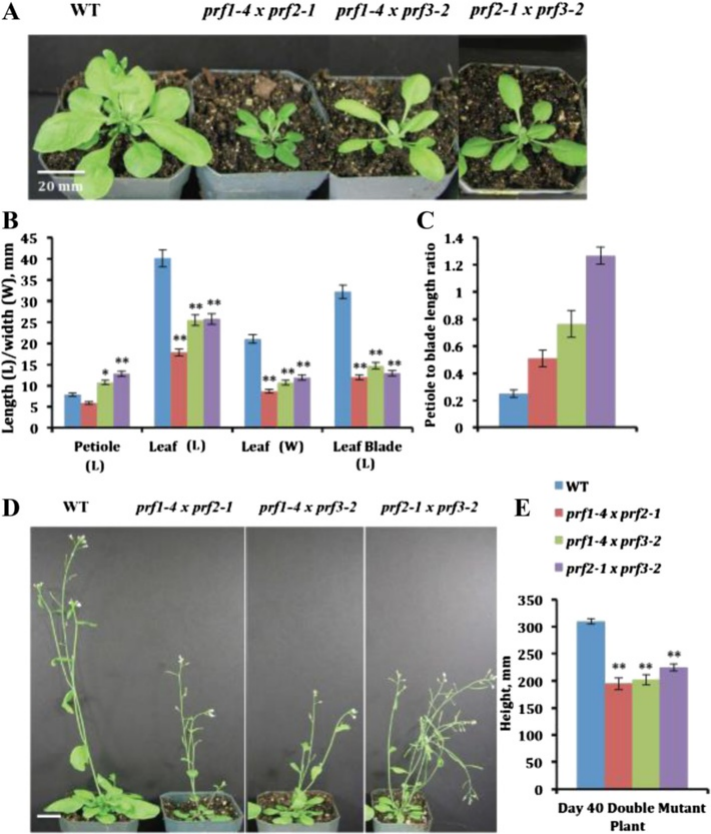
Morphological analysis of vegetative profilin double mutants. **a** Visualization of morphological phenotypes observed for profilin double T-DNA mutants. Pictures were taken at 4 weeks (4w) after seed germination. **b** Petiole length, leaf length, leaf width, and leaf blade length for double vegetative PRF T-DNA mutants. **c** Petiole to leaf blade length ratio for double PRF T-DNA mutants. **d** Pictures of double mutant plants showing morphological phenotypes at 5 weeks (5w). **e** Mature plant height for double mutants. Leaf measurements were taken on day 28 (4w, *n* = 52 for each measurement), while plant height measurements were taken on day 40 (~5 ½ w, *n* = 30). All measurements are in mm. Error bars represent +/- 1 SD. **p value <0.001, *p < 0.05

In order to assess both functional redundancy and quantitative effects of the vegetative profilins in *Arabidopsis* development, three double T-DNA mutants were generated: *prf1-4 prf2-1*, *prf1-4 prf3-2*, and *prf2-1 prf3-2.* The phenotypes shown in Fig. 3 make it clear that all three double homozygous mutants exhibit even stronger and more distinct developmental phenotypes than any single PRF1, PRF2, or PRF3 defective plant. The double mutant plants that are noticeably smaller than wild-type (Fig. 3a) had leaves that are remarkably shorter in total length, blade length, and width than the wild type (Fig. 3b and c) or the single mutants. These defects are seen throughout development (Fig. 3a and c). Interestingly, double mutants containing the *prf3-2* allele show longer petioles (Fig. 3b and c, see the next section). The double mutants are also shorter in overall plant height (Fig. 3e).

**Fig. 4:**
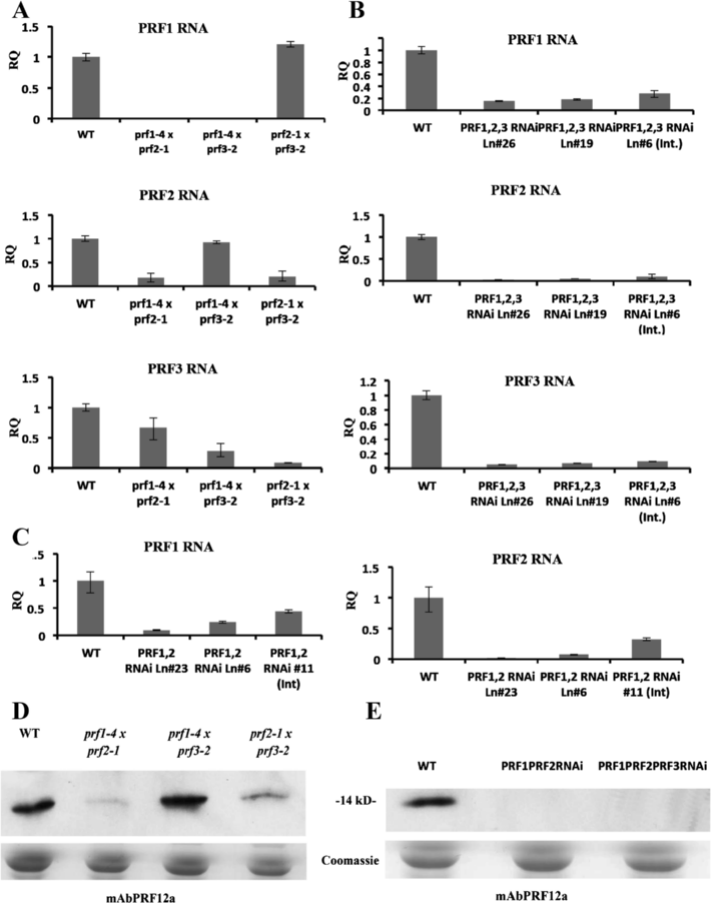
qRT-PCR data and western blot analysis for double and triple mutant/ RNAi lines. *Transcript expression (* ***a*** *,* ***b*** *,* & ***c*** *)* **a** qRT-PCR data for T-DNA double mutants. Each graph shows the RQ of *PRF1, PRF2,* or *PRF3* expression levels for each of the T-DNA double mutants. **b** qRT-PCR data for *PRF1PRF2PRF3*-RNAi lines (three lines shown). Each graph shows the RQ of *PRF1, PRF2*, or *PRF3* expression levels for each of the *PRF1PRF2PRF3*-RNAi lines. **c** qRT-PCR data for *PRF1PRF2*-RNAi lines (three lines shown). Each graph shows the RQ of either *PRF1* or *PRF2* expression levels for each of the *PRF1PRF2*-RNAi lines. Error bars represent +/- 1 SD. *Protein expression (* ***d & e*** *)* **d** Western analysis of protein levels in profilin double mutants (all three combinations) using the PRF1 and PRF2 specific monoclonal antibody mAbPRF12a. **e** Western analysis of *PRF1PRF2*-RNAi and *PRF1PRF2PRF3*-RNAi lines using the PRF1 and PRF2 specific monoclonal antibody mAbPRF12a. Coomassie stained gels showing rubisco protein expression are located beneath each blot to show equal loading across lanes. All samples were taken from 4w old leaf tissue

qRT-PCR and western blot analysis (with mAbPRF12a) again revealed that these mutants had very little to no detectable profilin RNA and even more greatly reduced profilin protein expression (Fig. 4a and d). Notice how the *prf1-4 prf3-2* double mutant had the strongest profilin expression among the three double mutants tested (Fig. 4d), which is in agreement with PRF2 being the most highly expressed member of the gene family. *prf1-4 prf2-1* had the lowest profilin expression (Fig. 4d), consistent with known expression levels for these two genes, and therefore produced the most drastic developmental phenotypes. *prf2-1prf3-2* plants also have a faint profilin band, possibly representing PRF1 protein. These data suggested that unlike *Arabidopsis* vegetative actin ACT7, which is up-regulated in response to deficiencies in ACT2 and ACT8, none of the vegetative profilins were significantly up-regulated in response to profilin deficiency.

**Fig. 5:**
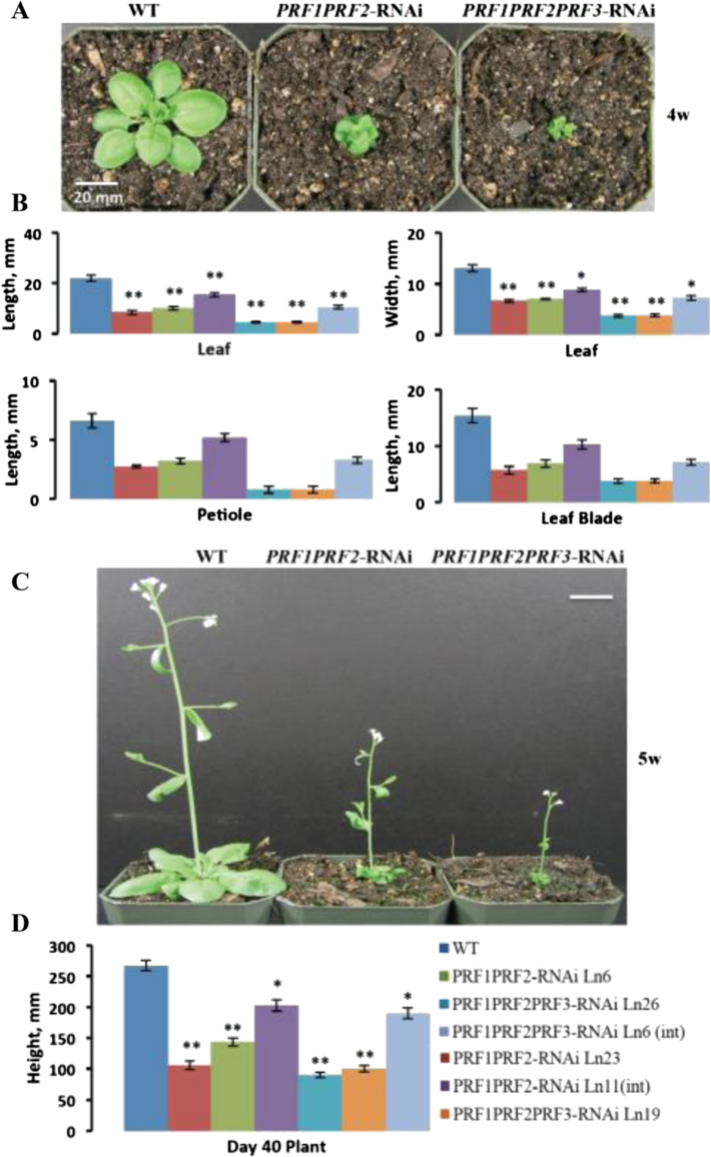
Morphological analysis of PRF double and triple RNAi lines. **a** The morphology of PRF double and triple RNAi lines (lines silenced for *PRF1* and *PRF2* and for *PRF1-3*, respectively) were examined 4 weeks (4w) post-germination. **b** Leaf length, leaf width, petiole length, and leaf blade length for PRF double and triple RNAi lines. **c** PRF double and triple RNAi plants show severe morphological phenotypes at 5 weeks (5w). **d** Mature plant height for double and triple RNAi lines. Leaf measurements were taken on day 28 (4w) during development (*n* = 52), while plant height measurements were taken on day 40 (~5 ½ w, *n* = 30). All measurements are in mm. Error bars represent +/- 1 SD. **p value <0.001, *p < 0.05

Based on previous studies that silenced four late pollen actins and four Actin Depolymerizing Factors (ADFs) by stacking four different 100 bp 3′-UTR sequences in the stem of a stem-loop RNA interference construct [29, 30], we used simplified construct designs (described above) to silence PRF1 and PRF2 (*PRF1 PRF2*-RNAi), as well as PRF1, PRF2, and PRF3 (*PRF1 PRF2 PRF3*-RNAi), simultaneously (see Methods). Based on qRT-PCR analysis of profilin transcript levels, we selected independent strongly silenced transgenic lines (#23 and #6 for *PRF1 PRF2*-RNAi, #26 and #19 for *PRF1 PRF2 PRF3*-RNAi) and intermediately silenced lines (#11 for *PRF1 PRF2*-RNAi, #6 for *PRF1 PRF2 PRF3*-RNAi) for detailed analyses. The strongly silenced lines were severely dwarfed throughout development, with the triple RNAi epiallele showing much more drastic phenotypes than the doubly silenced line or any of the double mutants (Fig. 5). qRT-PCR and western blot analysis of these lines show minute levels of RNA and no detectable protein expression (Fig. 4b, c, e). The dwarf *PRF1 PRF2 PRF3*-RNAi plants had fewer siliques that produced hardly any seeds, and the plants were significantly shorter than any of the other single or double mutant lines (Fig. 5d). These results indicated that when *Arabidopsis* plants were deficient in all three vegetative profilins there appears to be a quantitative genetic effect leading to severely dwarfed and less fertile plants. A wide range of tissues and organs were not fully developed (see below for example lateral root and leaf epidermal cell development). Interestingly, the double *PRF1 PRF2*-RNAi line exhibited slightly more radical phenotypes than the p*rf1-4 prf2-1* double mutant. Perhaps this is due to a low but barely detectable level of PRF1 expressed in prf1-4 allele. However, by looking at the western blot data (Fig. 4d-e) we saw that the *PRF1 PRF2*-RNAi line had even less protein than the *prf1-4 prf2-1* double mutant, which explains the more severe phenotypes.

#### PRF3 deficient plants exhibit slightly elongated petioles

**Fig. 6:**
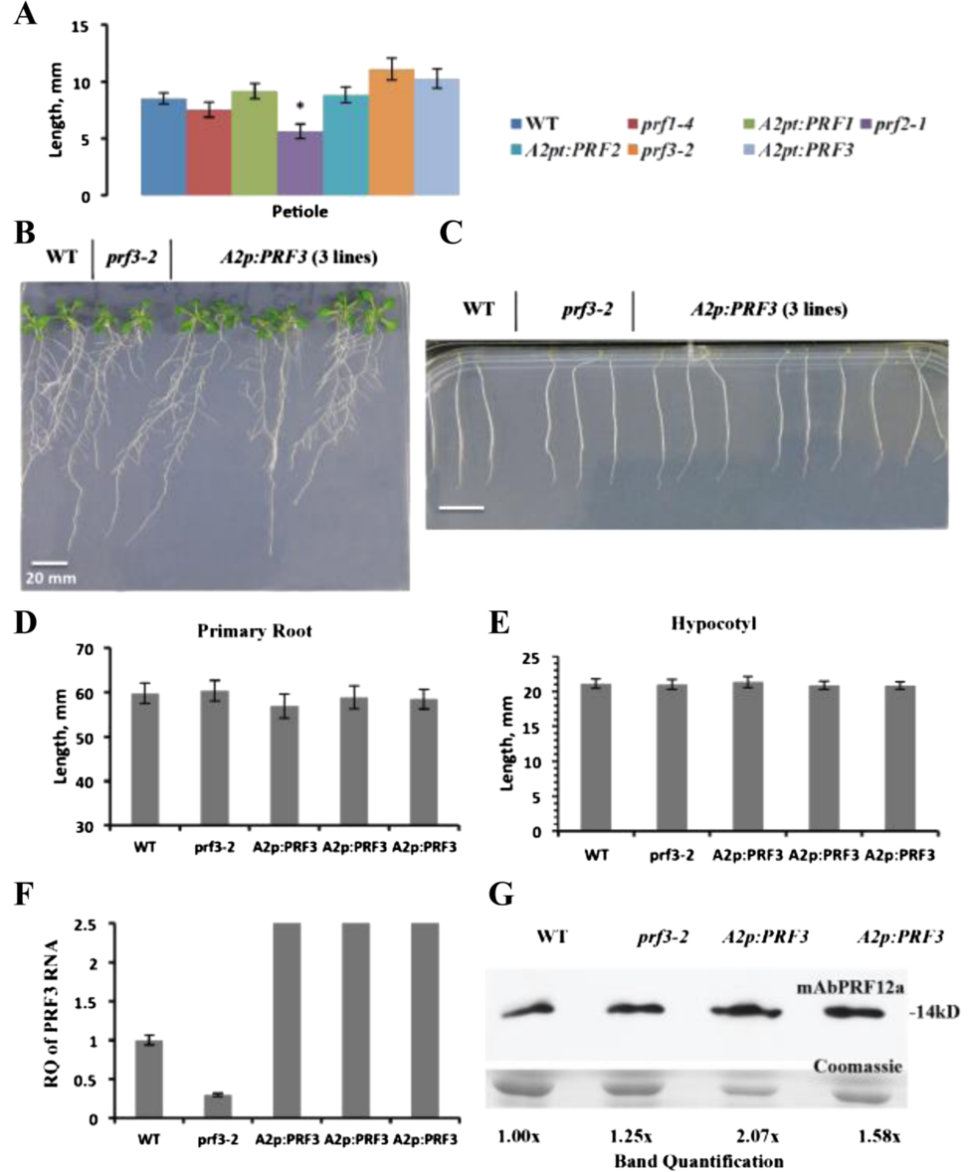
PRF3 knockout displays elongated petioles, while PRF3 overexpression results in no phenotypic effects. **a** Petiole length for vegetative PRF T-DNA mutant *prf3-2* and its complement lines. **b** Day 14 primary root length comparison between WT, *prf3-2*, and three independent *A2p:PRF3* overexpression lines. **c** Day 10 hypocotyl length comparison between WT, *prf3-2*, and three independent *A2p:PRF3* overexpression lines. Seedlings were grown under dark conditions. **d** Quantification of primary root length measurements from lines pictured in (**b**). **e** Quantification of hypocotyl length measurements from lines pictured in (**c**). **f** qRT-PCR data on the relative quantity of PRF3 RNA expression for WT, *prf3-2*, and three independent *A2p:PRF3* overexpression lines. **g** Western blot analysis for WT, *prf3-2*, and two independent *A2p:PRF3* overexpression lines. Western blot bands were quantified using the ImageJ software. All samples were taken from 4w old leaf tissue. Leaf measurements (a) were taken on day 28 (4w) during development, primary root length measurements (d) were taken on day 15, and hypocotyl length measurements (e) were taken on day 10. All measurements’ are in mm. Leaf measurements were generated with a sample of *n* = 52, while root and hypocotyls measurements have an *n* = 30. Error bars represent +/- 1 SD. *p value < 0.05

While PRF3 deficient plants did not seem to display the strongly dwarfed leaf phenotype similar to plants deficient in PRF1 or PRF2, they did exhibit elongated petioles compared to WT (Fig. 6a). The elongated petiole phenotype can be seen in all plant lines that have the PRF3 gene knocked down (*prf3-2*, *prf1-4* p*rf3-2*, and *prf2-1 prf3-2*), with the exception of *PRF1 PRF2 PRF3*-RNAi plant lines whose leaves and petioles were so dwarfed that this difference was not statistically significant (Figs. 3b, 5b, and 6a). Previous experiments using *PRF3* promoter-*GUS* fusion constructs confirmed that PRF3 was being expressed in petioles [31]. Our data suggest that PRF3 may play a distinct role in petiole development.

#### PRF3 overexpression analysis

Multiple independent PRF3 overexpression lines were analyzed by growing them vertically on plates containing 0.5 MS salts and 1 % sucrose germination media, yielding no phenotypic result (Fig. 6b). Three independent *A2p:PRF3* overexpression lines showed 8 to 20-fold increases in PRF3 transcript expression (Fig. 6f). Two lines examined, #1 and #3, with immunochemical staining of a Western blot with mAbPRF12a monoclonal antibody, showed about 1.6 and 2x higher levels of total profilin over WT (Fig. 6g). Considering that this antibody recognizes PRF3 more weakly than PRF1 or 2, these Western data suggest the actual levels of profilin protein may be significantly higher in these lines. Despite previously published data indicating that the overexpression of PRF3 causes stunted roots [31]; we saw no related phenotype in our PRF3 overexpression lines (Fig. 6b). In addition, no effects were seen on hypocotyl development in PRF3 overexpression plants (Fig. 6c). These contrasting phenotypic results could be due to differences in how PRF3 was overexpressed. PRF3 RNA expression levels in these lines were demonstrated using qRT-PCR (Fig. 6f). Western blot analysis was unable to clearly determine the extent of PRF3 protein present, since PRF3 represents such a small part of total profilin expression, and because mAbPRF12a reacts stronger with the more highly expressed PRF1 and PRF2 proteins than PRF3 (Fig. 6g). For this reason we rely more on our qRT-PCR analysis for gauging PRF3 levels. A complete list of phenotypic measurements for all plant lines is presented in Additional file 3: Table S1.

#### Vegetative profilins are essential to lateral root initiation

**Fig. 7:**
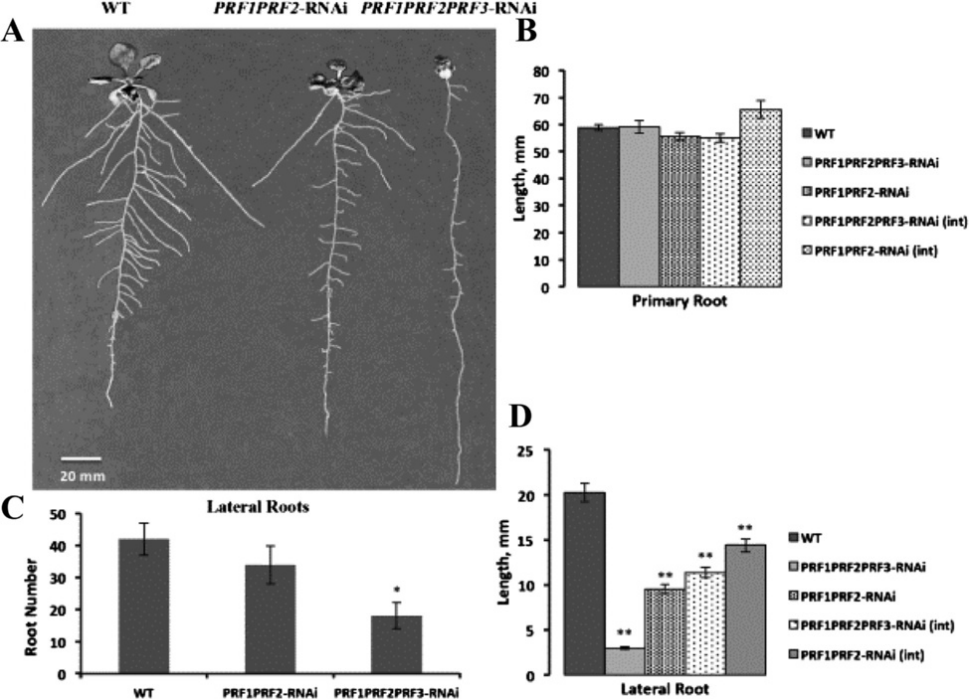
Vegetative profilin double and triple RNAi lines show defects in lateral root formation and growth. **a** Visualization of defects in root development for PRF double and triple RNAi lines. **b** Quantification of primary root length. **c** Quantification of the number of lateral roots formed/ initiated. **d** Quantification of lateral root length. Pictures and measurements were taken on day 15 of development. Sample size was 30 (*n* = 30) and error bars represent +/- 1 SD. **p value <0.001, *p < 0.05

We grew all mutant and epiallele plant lines vertically in plates on germination media containing 0.5 MS salts and 1 % sucrose to look for defects in root growth. Most single profilin-deficient lines showed no significant root growth and lateral root formation phenotypes (not shown). However, the RNAi lines lacking PRF1 and PRF2, the two most highly expressed profilins, had normal primary roots (Fig. 7a and b), but the lateral roots were slightly shorter than wild type as shown in Fig. 7a and d. However, multiple *PRF1 PRF2 PRF3*-RNAi plant lines revealed severe lateral root phenotypes. They formed primary roots of normal length, but showed drastic differences in their lateral root initiation and architecture (Fig. 7a, b, c). They not only produce a lower numbers of lateral roots (Fig. 7c), but these lateral roots were also much shorter (Fig. 7d), indicating that there could likely be a problem in cell elongation among these lateral roots. Root architecture of the intermediately silenced *PRF1 PRF2*-RNAi and *PRF1 PRF2 PRF3*-RNAi epialleles revealed intermediate phenotypes that appear proportional to PRF expression levels (Additional file 4: Figure S3). While primary root length appears slightly longer in the two intermediately silenced *PRF1 PRF2*-RNAi lines shown, they were not statistically significant (not shown).

## Discussion

### Vegetative profilins effect normal leaf and inflorescence development

The *Arabidopsis thaliana* genome encodes a five-member profilin gene family, producing three vegetative and two reproductive protein variants. We have focused on the functional consequences of knocking out or efficiently knocking down the three vegetative gene members both individually and in combinations. Single T-DNA insertion mutants, *prf1-4* and *prf2-1,* showed very similar phenotypic effects, with plants showing defects in normal rosette leaf morphology as well as inflorescence development, leading to shorter overall plant height for these mutants (Fig. 1). The inflorescences of these mutants were thinner and weaker than WT and not stable enough to stand up on their own. This suggests that there are structural deficiencies in these mutant tissues. Since profilin is thought to be responsible for shuttling monomeric actin to promote filament formation, perhaps lower profilin levels is inhibiting or slowing the formation of actin-filaments at the expanding edges of cells, resulting in a lack of appropriate cell expansion or elongation in these tissues. Interestingly, the *prf3-2* mutant showed no significant inflorescence phenotypes (Fig. 1).

Profilin promoter-reporter*-*constructs show that all three of these profilin promoters are expressed in the same tissues throughout the plant [31, 32], so it is somewhat surprising that similar phenotypes are not seen in the *prf3-2* mutant. One must remember that PRF1 and PRF2 are significantly more highly expressed than PRF3 [26], which may explain why the lower expressed PRF3 does not have such obvious developmental defects. This suggests there must be some functional redundancy for PRF3 among these other vegetative profilins.

While the rosette leaf and inflorescence phenotypes were absent from *prf3-2*, this mutant did appear to show effects on petiole development. In particular, plants lacking PRF3 showed slightly elongated petioles compared to WT (Fig. 6), indicating that PRF3 may be specifically required for proper petiole formation. Perhaps the PRF3 variant is responsible for controlling the proper spatial sequestering of actin monomers in petioles, thereby guiding normal petiole development. Alternatively, there could be greater stability of the PRF3 transcript or protein in petiole, increasing its importance relative to PRF1 and PRF2. Further studies on PRF3 deficiencies will be necessary in order to fully understand all of the specific functions of PRF3 in petiole and overall plant development. PRF3 overexpression lines were recently analyzed and show defects in seedling development, in particular, stunted primary root and hypocotyl length [31]. Both our lab’s work and theirs were performed using the *Arabidopsis* Columbia ecotype. However, after constructing PRF3 overexpression lines using the strong constitutive ACTIN2 promoter, and performing qRT-PCR and western analysis to confirm the overexpression of PRF3 RNA and protein, these phenotypes were not detected (Fig. 6b-e). In particular, we did not see any significant deviation from WT primary root and hypocotyl length (Fig. 6b-c). We are uncertain as to what to conclude from these two conflicting results.

It is worth noting again that we were unable to establish a successful, clean RNAi line that only targets PRF3 using a plethora of different methods. This is confusing since we were able to establish the triple knockdown line (*PRF1 PRF2 PRF3*-RNAi) with no issues. The fact that the portion of the triple RNAi construct targeting PRF3 was located at the base of the large, inverted stem loop structure suggests that the reason the single *PRF3*-RNAi did not work was due to steric hindrance. Regardless, single mutant analysis has revealed that when each of these genes is knocked out, abnormalities arise in plant development and these phenotypes are corrected when complemented by the corresponding overexpression construct.

### Knocking out multiple vegetative profilins leads to compounded phenotypic defects

After seeing that individual single gene mutants gave rise to noticeable phenotypes, we developed novel plant lines where multiple PRF genes were knocked out. This is the first reported analysis of multiple profilin deficiencies. We saw the same developmental phenotypes as the single mutants, but they were significantly more extreme. While *prf1-4* and *prf2-1* had similar leaf and inflorescence defects, the *prf1-4 prf2-1* double mutant gave rise to plants with statistically smaller leaves than the respective single mutants (Fig. 3). However, the *prf1-4 prf2-1* double mutant did not lead to plants that were significantly shorter in overall plant height than the single mutants. This was unexpected, because it is hard to propose how the plant actin cytoskeleton can support normal inflorescence growth with abnormally low levels of profilin. However, the doubly deficient *PRF1 PRF2*-RNAi lines did demonstrate a significant drop in plant height as well as leaf size (Fig. 4). This difference between mutants and RNAi lines could be attributed to the fact that the *prf2-1* insertion is in the promoter region upstream of the transcriptional start site leading to some leakiness when crossed with another mutant. Regardless, there is an agreement between the two approaches that by knocking out the two most highly expressed vegetative profilins, you see the most dramatic leaf and inflorescence phenotypes, yet the plants are still viable.

While the *prf1-4 prf2-1* mutant showed the most compounded developmental defects, we saw a combination of single mutant phenotypes in the *prf1-4 prf3-2* and the *prf2-1 prf3-2* double mutants. These plants both displayed dwarfed leaves, shorter, less stable inflorescences, and elongated petioles (Fig. 3). This was fortunately exactly what we expected. This would indicate that while PRF1 and PRF2 are playing major roles in rosette and inflorescence development, PRF3 must be involved in the proper development of petioles. Based upon overall expression levels, it makes sense that the much lower expressed PRF3 seems to have evolved to function specifically in the assistance of petiole development, while PRF1 and PRF2 serve to function in multiple tissues. In addition, because the PRF1 and PRF2 deficient plants exhibit very similar phenotypic effects, this suggests the possibility of their being partial functional redundant. However, since the single mutants each have strong phenotypes, we suspect there is also a quantitative genetic effect.

### PRF1 PRF2 PRF3-RNAi plants show the most drastic dwarfed phenotypes and exhibit defects in lateral root growth and formation

To further dissect the role vegetative profilins are playing in *Arabidopsis* development and examine possible quantitative genetic effects, we created an RNAi construct using a modification of a published method [29, 30], that silences all three profilin genes simultaneously, *PRF1 PRF2 PRF3*-RNAi. The construct offers the advantage of being smaller, requiring less effort and/or less expense than previous methods, and produces a stem loop silencing RNA with only four ‘A’ residues in the loop. Molecular characterization has shown that the phenotypically most severe plants did not express any detectable vegetative profilin protein (Fig. 4e). They were dramatically dwarfed in the size of all organs and structures (Fig. 5). It appears that when the vegetative profilin pool is almost completely depleted, plants are unable to fully form many of its above ground tissues and organs. This dramatically dwarfed phenotype is indicative of defects in cell number, expansion, and elongation. Surprisingly, these plants are not fully sterile; they did produce some seeds, but due to their sickening health they were very difficult to genetically manipulate.

The overwhelming above ground phenotypes in these plants prompted a more detailed analysis into their root development. It was recently reported that at root tips, actin polymerization is facilitated by the Actin Related Protein 2/3 (Arp2/3) complex and profilin through interactions with phosphatidylinositol 4,5-bisphosphate [33], thereby implicating profilin with proper root elongation. All single PRF mutants yielded no effects on root development. When PRF1 and PRF2 were knocked down, we see slight root defects. When all three PRFs were knocked down we observed major deficiencies in the formation of the overall root architecture (Fig. 7a). In particular, *PRF1 PRF2 PRF3*-RNAi plants lacked the ability to initiate significant numbers of lateral roots and extend them. While it is surprising that primary root growth appears unaffected in plant lines with undetectable levels of profilin, defects in lateral root growth and formation are abundantly clear. This suggested that the total amount of profilin needs to be at some minimal level in order to properly initiate lateral root formation. This would indicate that there was functional redundancy among the vegetative profilin gene family, and that having any of these three profilins is sufficient for proper lateral root initiation and growth. Perhaps once overall vegetative profilin protein levels reach a certain threshold, the cells conserve what is present and only initiate cell elongation in certain tissues and organs (possibly those more essential to development or survival). This result begs the question- without measurable profilin, what actin binding protein(s) are controlling the interaction between the actin monomer pool and F-actin? Because we do see a milder root architecture and lateral root growth phenotype in our intermediately silenced *PRF1 PRF2 PRF3*-RNAi line (Additional file 4: Figure S3); we believe that this is a result of a quantitative genetic effect.

### Lowering PRF concentrations may lead to altered cytoskeletal dynamics

Data from a variety of studies suggests that profilin may be functioning in actin polymerization and/or depolymerization, and it seems likely that profilin is doing both. This is true for another class of small ABPs, the Actin depolymerizing factors (ADFs), which are known to stabilize and/or sever F-actin filaments in a concentration dependent manner [34, 35]. In the presence of profilin, filament elongation occurs exclusively at the barbed ends, while elongation at the pointed ends appears inhibited. In the absence of profilin, elongation appears to occur at the same rate on barbed and pointed ends suggesting that profilin could be essential for directional filament elongation [16]. Furthermore, X-ray structure analysis has shown that profilin is required for the nucleotide binding pocket of actin to remain open and stable [36]. This conformation facilitates ADP to ATP nucleotide exchange in actin monomoers, and hence, is a crucial intermediate in the actin depolymerization/polymerization cycle, thereby linking profilin to both actin polymerization and depolymerization [37].

Recent studies have found that the slow release of inorganic phosphate (Pi) from the barbed end of actin filaments is linked to an increase in the rate of filament disassembly, and is further accelerated by profilin [38]. This is evidence that profilin facilitates the disassembly of actin filaments. Other studies have shown that the overexpression of profilin by microinjection inhibited pollen tube elongation [39, 40]. Yet, in order for a cell to expand or elongate there must be a rapid treadmilling (turnover) of actin, which is facilitated through ABPs like profilin and ADF [41]. Altogether, these data support the view that profilin is involved in both the polymerization and depolymerization of actin filaments, and are neccessary for normal plant cell expansion or elongation.

Our original hypothesis was that decreasing profilin levels would act to free up more actin monomers to form F-actin filaments, which would in turn lead to more rapid cell elongation. However, we are observing the opposite result in plants that are severely deficient in PRF1 and PRF2. Based on the previous findings and the results presented here, we suggest a model in which decreasing the profilin pool by small amounts might lead to a sensory signal that tells the cell to start elongating quickly, whereas major reductions of profilin will lead to a physical arrest in cell elongation. This quantitative genetic effect of lowering profilin pool concentrations suggest that if actin monomers are unable to bind profilin, there will not be enough profilin-actin complexes being properly sequestered to the cell periphery to promote appropriate cell elongation. Furthermore, the lack of profilin would lead to defects in actin treadmilling, which is required for cell elongation. This would inevitably cause arrest in actin filament protrusion leading to plants with smaller leaves, as was seen in our profilin mutants. This model agrees with our findings that there is a direct correlation between the number of profilin genes that were knocked out, and the severity of the dwarfed plant phenotype. In short, there appears to be a complex “bimodal” relationship between profilin concentrations and the quality of cell elongation phenotypes. Further experiments looking into actin filament organization and turnover in PRF deficient plants will be needed to establish a concrete mechanism for PRFs role in actin dynamics.

## Conclusions

In conclusion, we have demonstrated that vegetative profilins play an essential role in *Arabidopsis* development and the regulation of the actin cytoskeleton. Dramatic decreases in vegetative profilin gene expression produce more compounded phenotypes, suggesting that there is a direct correlation between profilin concentrations and defects in development. While the model presented herein serves to explain the phenotypic effects of lowering profilin levels, the exact mechanisms still need to be clarified in future studies. The fact that slight reductions produce a very different effect from large reductions in profilin levels suggests there is a need for a more detailed dissection of these mechanisms. This paper analyzed profilins’ role in promoting proper cell elongation, but additional research is needed to examine their roles in signal transduction, intracellular transport, and communication. We suggest that a systems biology approach may be needed to dissect out how all of these processes are interacting with each other through a profilin intermediate.

## Methods

### Plant materials and growth conditions

All *Arabidopsis thaliana* seeds were of the Columbia (Col) ecotype. Wild-type, mutant, and transgenic seeds were grown in conditions and media described previously [42, 27, 43]. T-DNA insertion lines were obtained from the *Arabidopsis* Biological Resource Center (ARBC Ohio St. University). *prf1-4* (GK_614F01) and *prf3-2* (GK_055A02) were from the Gabi Kat mutant collection, while *prf2-1* (SALK_129071) was generously provided to us from Dr. Brad Day (Michigan St. University), and is derived from the SALK mutant collection. T-DNA mutant lines were cleaned up by backcrossing to WT-Col, allowing heterozygotes for the insertion to self-pollinate, and then repeating the process for a second and third time to ensure that these lines are free of other T-DNA insertions. These plants were screened each generation for the presence of their respective mutant alleles by PCR using methods previously described [44] and the following sets of mutant Left Border (LB) and WT primers: *prf1-4*, PRF1_WT_S (5′-TAGACCATTAGTCTATTGTGAGAT-3′), Prf1-4_GK_LB (5′- CGTCGGAGAATTCAGTACTCG-3′), and PRF1_WT_AS (5′-TTCGCCACCGAGAAATAGTCCGGTT-3′), *prf2-1*, PRF2_WT _S (5′-ATCGACTTTCACACAAAACAT-3′), Prf2-1_SALK_LB (5′-GCAATTAGCTTCAACCGACTG-3′),and PRF2_WT_AS (5′-TTGCCTTCGACCTCGCACATGAGAT-3′), *prf3-2*, PRF3_WT_S (5′-AGATGAGGGCCTTATAATGGA-3′), Prf3-2_GK_LB_S (5′- ATCATCGATCGGCTCATATTG-3′),and PRF3_WT_AS (5′-GTAGTCGGTATAGAAATA-3′). DNA for PCR was extracted using the REDExract N-Amp Plant PCR Kit (Sigma-Aldrich). Following confirmation via PCR, clean mutant lines were sent off for DNA sequencing to confirm the exact location of the insertions. *prf1-4* had an insertion 74 bp upstream of the second exon in the first intron, *prf2-1* had an insertion 113 bp upstream of the translational start site in the promoter, and *prf3-2* had an insertion 15 bp from the end of the first exon (Fig. 1b). All plants were grown at 22 °C with 16-h days/ 8-h nights.

#### Generation of double mutants

Double mutants were then generated through the following plant crosses between the individual T-DNA mutants: *prf1-4/ prf1-4* pollen crossed with emasculated *prf2-1/ prf2-1* (*prf1-4 prf2-1*), *prf1-4/ prf1-4* pollen crossed with emasculated *prf3-2/ prf3-2* (*prf1-4 prf3-2*), *prf2-1/ prf2-1* pollen crossed with emasculated *prf3-2/ prf3-2* (*prf2-1 prf3-2*). F1 progeny were screened by PCR for the presence of both alleles (using primers above), and then allowed to self-pollinate. PCR was used to check F2 progeny displaying the dwarfed leaves phenotypes for the presence of both mutant alleles and the absence of both wild-type alleles.

#### Simplified construction of RNAi transgenes

Single, double, and triple RNAi constructs were designed based on previously described methods [29, 30] with an important simplification. Previous constructs used a large 1400 bp petunia intron to separate the forward and reverse facing sequences and RNAi gene constructions required going through multiple rounds of overlapping PCR or a multistep cloning process to make the assembly. Instead, we used a 79 bp Actin2 intron flanked by two “A” residues on either side and had it synthesized by GenScript (Piscataway, NJ). This design allowed for a much smaller gene construct to be assembled. The constructs consisted of 100 (*PRF1*-RNAi), 200 (*PRF2L*-RNAi and *PRF1 PRF2*-RNAi), or 300 (*PRF1 PRF2 PRF3*-RNAi) bp inverted repeats (depending on how many genes being targeted) separated by the “A” residues and the 79 bp Actin2 intron, all under the control of the Actin2 promoter terminator (A2pt) [19]. The advantages of these constructs are that they were inexpensively synthesized as 283, 483, and 683 bp sequences, respectively, and were cloned in one step into an expression vector. Once the intron was removed, we were left with a stable “AAAA” loop connected to the RNA stem consisting of the inverted repeats that hybridize to the first 100 bp (200 bp for *prf2-*RNAi) of the 3′-UTRs of their corresponding profilin target genes. *PRF2*-RNAi required a longer inverted stem of 200 bp in order to achieve sufficient silencing of PRF2.

Complementation Constructs were made by cloning full-length PRF1, PRF2, and PRF3 cDNAs under the control of the A2pt construct, as described in [19]. This ensured the proper expression in the appropriate tissues. Fimbrin-GFP reporter constructs (*35S:GFP-FABD2*); previously described in [32] were transformed into our WT and *PRF1 PRF2*-RNAi plants to allow for visualization of actin filaments. The *35S:GFP-FABD2* construct consists of GFP fused to the C-terminal half of *Arabidopsis* Fimbrin1. For our constructs, we exchanged the hygromycin resistance marker for a Basta resistance marker. All transformations were performed with *Agrobacterium tumefaciens* strain C58C1 using the floral dip method [45, 46].

#### Leaf, root, and plant measurements

All leaf measurements were taken using a standard metric ruler on day 28 of plant development (i.e., 28 days after seed germination on soil). For each measurement, a total of 52 rosette leaves (largest two leaves per plant on 26 plants) were analyzed from WT, mutant, complement, and RNAi lines. Plant height measurements were taken on day 40 of development after laying plants flat on the bench and measuring the length from the base of the rosette to the top of the inflorescence. For each measurement, a total of 30 plants were analyzed for WT, mutant, complement, and RNAi lines. Root quantifications were made on day 15 of development using a standard metric ruler for measuring the length or by counting the number of lateral roots initiated. For each measurement, a total of 30 roots were analyzed for WT, *PRF1 PRF2*-RNAi, *PRF1 PRF2 PRF3*-RNAi, *prf3-2*, and *A2p:PRF3* overexpression lines. To measure the hypocotyls, seeds were grown vertically in dark growth conditions with measurements taken on day 10. All measurements were taken to the nearest 0.1 mm. Graphs of resulting data were constructed in Excel (Microsoft).

#### qRT-PCR RNA analysis

RNA was isolated, treated, and cDNA was made from leaf tissues of wild-type and various transgenic or mutant plants as previously described [20]. cDNA populations were analyzed using the following qRT-PCR primers: Ubiquitin10 (Ubiq10) was the endogenous control, Ubiq10_Sense (5′-AGAAGTTCAATGTTTCGTTTCATGTAA-3′) and Ubiq10_Antisense (5′-GAACGGAAACATAGTAGAACACTTATT-3′), PRF1, PRF1_3utr_Sense (5′-TCTCCTTCGTTACCGAGTTTGAG-3′) and PRF1_3utr_Antiense (5′-ACTCAATACATATGGAGAAAAAAGAT-3′), PRF2, PRF2_3utr_Sense (5′-CTGCCATGTATTGTGATTTGATTG-3′) and PRF2_3utr_Antiense (5′-GAGAGGATCAAAACCATAACAAATAT-3′), PRF3, PRF3_3utr_Sense (5′-GTGTCGTGAGAGAAAAACTATTCGAT-3′) and PRF3_3utr_Antiense (5′-CCCCAAGATCCATCACAAGGT-3′). All primer sets were designed to detect the 3′-UTR of their respective genes, thus ensuring distinct specificity and that primers were downstream of all T-DNA insertions. Reactions were performed on an Applied Biosystems 7500 real-time PCR system using SYBR Green detection chemistry (Applied Biosystems) as described previously [47]. In all experiments, the delta-delta-Ct algorithm (2^−(ddCT)^ method) [48] was used to detect the relative quantification of gene expression.

#### Western blot analysis

*Arabidopsis* protein samples were prepared by grinding 50 mg of frozen leaf tissue in liquid nitrogen and then extracted in 125 μL of extraction buffer containing 25 mM Tris–HCl, pH 7.5, 10 mM NaCl, 10 mM MgCl_2_, 5 mM EDTA, and a protease inhibitor cocktail (Roche Diagnostics; one tablet/10 mL). After 10 min centrifugation, the supernatant was mixed 1:1 with 2× Sodium Dodecyl Sulfate (SDS) sample buffer [49] and boiled for 5 min. ~15–20 μL were loaded per well (i.e., ~25 μg protein). Protein samples were then separated on 12 % SDS-PAGE gels and transferred to Immobilon transfer membrane (Millipore, Billerica, MA) by semi-dry blotting (Hoefer, San Francisco, CA). Membranes were blocked for 30 min in Tris-Buffered Saline and Tween 20 (TBST) (10 mM Tris–HCl pH 7.5, 150 mM NaCl, 0.05 % Tween 20) containing 20 % goat serum and 5 % dry milk, and then probed with the primary antibody that recognized a 13- to14-kD profilin band (mAbPRF1a or mAbPRF12a, see [19] at 0.5 mg/ml concentration for 1 h, and then washed thoroughly with TBST. Then membranes were probed with IgG-antimouse horseradish peroxidase-conjugated secondary antibody at a 1:2000 dilution in blocking solution for 30 min. The blots were washed again in TBST (3 x 5 min), treated with ECL detection solution (Amersham, Piscataway, NJ) for about 2 min and then exposed to the Hyperfilm ECL (Amersham, Piscataway, NJ). Western blot analysis was repeated at least twice for each experiment. Coomassie Brilliant Blue staining of duplicate gels was used to monitor the equal loading of proteins and to adjust loading if necessary. Quantification of bands was calculated using ImageJ (NIH), a Java-based image-processing program.

## Additional files

Additional file 1: Figure S1. Morphology of vegetative profilin single mutants. Visualization of the morphological phenotypes seen in profilin single T-DNA mutants at 2 weeks (2w), 3 weeks (3w), and 5 weeks (5w) post germination. A) WT, *prf1-4*, and *A2p:PRF1* complemented plants across development. B) WT, *prf2-1*, and *A2p:PRF2* complemented plants across development. C) WT, *prf3-2,* and *A2p:PR3* complemented plant across development. Additional file 2: Figure S2. Morphology and qRT-PCR analysis of transcript levels for vegetative PRF single RNAi lines. A) Morphological phenotypes of *PRF1*-RNAi and *PRF2*-RNAi lines across development at 4 weeks (4w) and 5 weeks (5w) post germination. B) Quantification of petiole length, leaf length, leaf width, and leaf blade length for *PRF1*-RNAi and *PRF2*-RNAi lines. C) Quantification of mature plant height for *PRF1*-RNAi and *PRF2*-RNAi lines. Leaf measurements were taken on day 28 (4w) during development (*n* = 52), while plant height measurements were taken on day 40 (~5 ½ w, *n* = 30). All measurements are in mm. D) qRT-PCR data representing the RQ of *PRF1* RNA for WT and *PRF1*-RNAi. E) qRT-PCR data representing the RQ of *PRF2* RNA for WT and *PRF2*-RNAi. Error bars represent +/- 1 SD. ***p* value <0.001, *p < 0.05. Additional file 3: Table S1. Summary of all phenotypic measurements for the various mutants and RNAi silenced plant lines examined. Additional file 4: Figure S3. Vegetative PRF double and triple RNAi lines that are only weakly silenced for profilin RNA expression show slight defects in lateral root development. Visualization of slight defects in root development for PRF double and triple RNAi lines with intermediate silencing (~40 % of WT levels). Pictures were taken 15 days after seed germination. Measurements can be seen in Fig. 7b and d.

## Abbreviations

ABPs: Actin Binding Proteins
ACT: Actin
PRFs: Profilins
G-actin: Globular actin
F-actin: Filamentous actin
T-DNA: Transfer DNA
RNAi: RNA interference
A2pt: Actin2 promoter and terminator
ADFs: Actin Depolymerizing Factors
Arp2/3: Actin Related Protein 2/3
Col: Columbia
LB: Left Border
Ubiq10: Ubiquitin10
2^−(ddCT)^: delta-delta-Ct algorithm
SDS: Sodium Dodecyl Sulfate
TBST: Tris-Buffered Saline and Tween 20
