# Supplementary figures and images for: Arabidopsis plants deficient in constitutive class profilins reveal independent and quantitative genetic effects
Source: BMC Plant Biol. 2015 Jul 11;15:177. doi: 10.1186/s12870-015-0551-0 (PMC4702419; doi:10.1186/s12870-015-0551-0)

**Additional file 1**

**Figure S1**

**
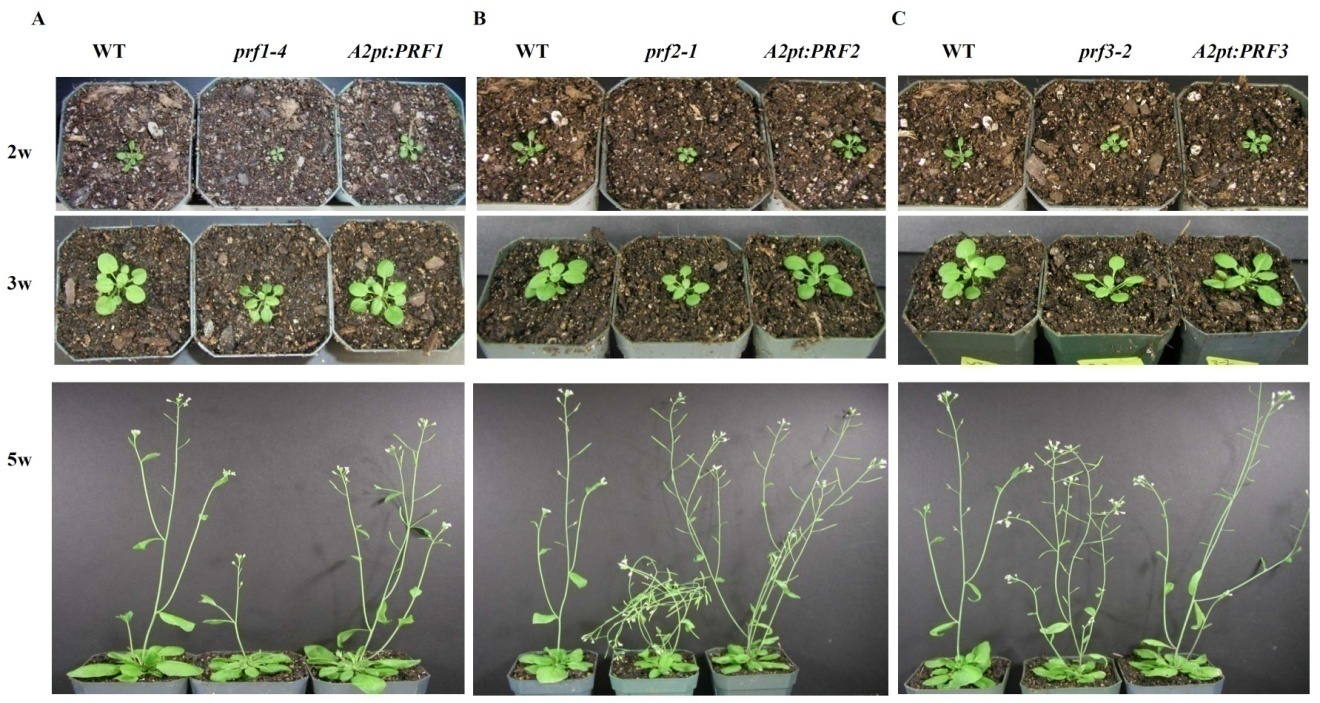
**

Supplement: Additional file 1: Figure S1. — Morphology of vegetative profilin single mutants. Visualization of the morphological phenotypes seen in profilin single T-DNA mutants at 2 weeks (2w), 3 weeks (3w), and 5 weeks (5w) post germination. A) WT, prf1-4, and A2p:PRF1 complemented plants across development. B) WT, prf2-1, and A2p:PRF2 complemented plants across development. C) WT, prf3-2, and A2p:PR3 complemented plant across development. [file 12870_2015_551_MOESM1_ESM.doc]
