# Supplementary material for: Arabidopsis plants deficient in constitutive class profilins reveal independent and quantitative genetic effects
Source: BMC Plant Biol. 2015 Jul 11;15:177. doi: 10.1186/s12870-015-0551-0 (PMC4702419; doi:10.1186/s12870-015-0551-0)
Supplement: Additional file 2: Figure S2. — Morphology and qRT-PCR analysis of transcript levels for vegetative PRF single RNAi lines. A) Morphological phenotypes of PRF1-RNAi and PRF2-RNAi lines across development at 4 weeks (4w) and 5 weeks (5w) post germination. B) Quantification of petiole length, leaf length, leaf width, and leaf blade length for PRF1-RNAi and PRF2-RNAi lines. C) Quantification of mature plant height for PRF1-RNAi and PRF2-RNAi lines. Leaf measurements were taken on day 28 (4w) during development (n = 52), while plant height measurements were taken on day 40 (~5 ½ w, n = 30). All measurements are in mm. D) qRT-PCR data representing the RQ of PRF1 RNA for WT and PRF1-RNAi. E) qRT-PCR data representing the RQ of PRF2 RNA for WT and PRF2-RNAi. Error bars represent +/- 1 SD. **p value <0.001, *p < 0.05. [file 12870_2015_551_MOESM2_ESM.doc]

**Additional file 2**

**Figure S2**
