# Supplementary material for: Arabidopsis plants deficient in constitutive class profilins reveal independent and quantitative genetic effects
Source: BMC Plant Biol. 2015 Jul 11;15:177. doi: 10.1186/s12870-015-0551-0 (PMC4702419; doi:10.1186/s12870-015-0551-0)
Supplement: Additional file 3: Table S1. — Summary of all phenotypic measurements for the various mutants and RNAi silenced plant lines examined. [file 12870_2015_551_MOESM3_ESM.doc]

**Additional file 3**

Table S1

|  | **Day 28 Petiole Length (mm)** | **Day 28 Leaf Length (mm)** | **Day 28 Leaf Width (mm)** | **Day 28 Leaf Blade Length (mm)** | **Petiole to Blade Length Ratio** | **Day 40 Plant Height (mm)** |
| --- | --- | --- | --- | --- | --- | --- |
| **WT (single T-DNA mutants)** | 8.97 | 35.65 | 17.91 | 26.67 | 0.417 | 309.42 |
| ***prf1-4*** | 7.54 | 19.34 | 9.93 | 11.80 | 0.664 | 204.77 |
| ***A2p:PRF1*** | 9.95 | 30.69 | 12.16 | 20.74 | 0.569 | 262.46 |
| ***prf2-1*** | 5.63 | 17.45 | 10.36 | 11.82 | 0.485 | 200 |
| ***A2p:PRF2*** | 10.85 | 34.57 | 13.52 | 23.71 | 0.526 | 258.85 |
| ***prf3-2*** | 11.11 | 28.53 | 13.35 | 17.41 | 0.668 | 238.35 |
| ***A2p:PRF3*** | 10.12 | 31.49 | 14.83 | 21.37 | 0.404 | 316.92 |
| **WT (RNAi lines)** | 9.63 | 30.38 | 16.40 | 20.75 | 0.472 | 301.08 |
| ***PRF1 RNAi*** | 5.42 | 15.75 | 9.91 | 10.33 | 0.540 | 165.44 |
| ***PRF2 RNAi*** | 5.64 | 16.66 | 11.20 | 11.01 | 0.520 | 207.08 |
| **WT (double T-DNA mutants)** | 7.92 | 40.12 | 21.07 | 32.21 | 0.250 | 309.53 |
| ***prf1-4 prf2-1*** | 5.88 | 17.87 | 8.66 | 11.98 | 0.509 | 195.00 |
| ***prf1-4 prf3-2*** | 10.77 | 25.50 | 10.70 | 14.72 | 0.764 | 202.05 |
| ***prf2-1 prf3-2*** | 12.84 | 25.82 | 11.91 | 12.98 | 1.269 | 225.05 |
| ***PRF1PRF2-* RNAi Ln#23** | 2.74 | 8.40 | 6.64 | 5.66 | 0.509 | 105.52 |
| ***PRF1PRF2-* RNAi Ln#6** | 3.21 | 10.08 | 6.99 | 6.87 | 0.477 | 143.28 |
| ***PRF1PRF2-* RNAi Ln#11 (int)** | 5.20 | 15.48 | 8.79 | 10.28 | 0.516 | 202.84 |
| ***PRF1PRF2PRF3-* RNAi Ln#26** | 0.78 | 4.55 | 3.70 | 3.78 | 0.211 | 89.76 |
| ***PRF1PRF2PRF3-* RNAi Ln#19** | 0.78 | 4.56 | 3.81 | 3.78 | 0.209 | 100.12 |
| ***PRF1PRF2PRF3-* RNAi Ln#6 (int)** | 3.29 | 10.43 | 7.25 | 7.14 | 0.465 | 190.28 |
