# Supplementary material for: Arabidopsis plants deficient in constitutive class profilins reveal independent and quantitative genetic effects
Source: BMC Plant Biol. 2015 Jul 11;15:177. doi: 10.1186/s12870-015-0551-0 (PMC4702419; doi:10.1186/s12870-015-0551-0)
Supplement: Additional file 4: Figure S3. — Vegetative PRF double and triple RNAi lines that are only weakly silenced for profilin RNA expression show slight defects in lateral root development. Visualization of slight defects in root development for PRF double and triple RNAi lines with intermediate silencing (~40 % of WT levels). Pictures were taken 15 days after seed germination. Measurements can be seen in Fig. 7b and d. [file 12870_2015_551_MOESM4_ESM.doc]

**Additional file 4**

**Figure S3**
